# *Galdieria sulphuraria*: An Extremophilic Alga as a Source of Antiviral Bioactive Compounds

**DOI:** 10.3390/md21070383

**Published:** 2023-06-28

**Authors:** Annalisa Ambrosino, Annalisa Chianese, Carla Zannella, Simona Piccolella, Severina Pacifico, Rosa Giugliano, Gianluigi Franci, Antonino De Natale, Antonino Pollio, Gabriele Pinto, Anna De Filippis, Massimiliano Galdiero

**Affiliations:** 1 Department of Experimental Medicine, University of Campania “Luigi Vanvitelli”, 80138 Naples, Italy; annalisa.ambrosino@unicampania.it (A.A.); annalisa.chianese@unicampania.it (A.C.); carla.zannella@unicampania.it (C.Z.); rosa.giugliano@unicampania.it (R.G.); anna.defilippis@unicampania.it (A.D.F.); 2 Department of Environmental, Biological and Pharmaceutical Sciences and Technologies, University of Campania “Luigi Vanvitelli”, 81100 Caserta, Italy; simona.piccolella@unicampania.it (S.P.); severina.pacifico@unicampania.it (S.P.); 3 Department of Medicine, Surgery and Dentistry “Scuola Medica Salernitana”, University of Salerno, 84081 Baronissi, Italy; gfranci@unisa.it; 4 Department of Biology, University of Naples Federico II, Complesso Universitario di Monte Sant’Angelo, 80126 Naples, Italy; antonino.denatale@unina.it (A.D.N.); antonino.pollio@unina.it (A.P.); gabriele.pinto@unina.it (G.P.)

**Keywords:** microalgae, extremophilic, antiviral, SARS-CoV-2, coronavirus, herpesvirus

## Abstract

In the last decades, the interest in bioactive compounds derived from natural sources including bacteria, fungi, plants, and algae has significantly increased. It is well-known that aquatic or terrestrial organisms can produce, in special conditions, secondary metabolites with a wide range of biological properties, such as anticancer, antioxidant, anti-inflammatory, and antimicrobial activities. In this study, we focused on the extremophilic microalga *Galdieria sulphuraria* as a possible producer of bioactive compounds with antiviral activity. The algal culture was subjected to organic extraction with acetone. The cytotoxicity effect of the extract was evaluated by the 2,5-diphenyl-2H-tetrazolium bromide (MTT) assay. The antiviral activity was assessed through a plaque assay against herpesviruses and coronaviruses as enveloped viruses and poliovirus as a naked one. The monolayer was treated with different concentrations of extract, ranging from 1 µg/mL to 200 µg/mL, and infected with viruses. The algal extract displayed strong antiviral activity at non-toxic concentrations against all tested enveloped viruses, in particular in the virus pre-treatment against HSV-2 and HCoV-229E, with IC_50_ values of 1.7 µg/mL and IC_90_ of 1.8 µg/mL, respectively. However, no activity against the non-enveloped poliovirus has been detected. The inhibitory effect of the algal extract was confirmed by the quantitative RT-PCR of viral genes. Preliminary chemical profiling of the extract was performed using ultra-high-performance liquid chromatography coupled to high-resolution mass spectrometry (UHPLC-HRMS), revealing the enrichment in primary fatty acid amides (PFAA), such as oleamide, palmitamide, and pheophorbide A. These promising results pave the way for the further purification of the mixture to explore its potential role as an antiviral agent.

## 1. Introduction

Widespread viral infections are a serious problem because of the potential emergence of resistance to available antiviral drugs and limited therapies for many viruses [1]. In the current pandemic era, with a clear need to identify novel antiviral treatments against emerging viruses, natural sources can be explored as producers of new pharmaceutical approaches. It is known that nature is an endless source of compounds with antimicrobial activity, and several research have shown that microalgae represent a valid solution to address the current challenges, both as antiviral and as dietary supplement. Additionally, microalgae are excellent candidates for genetic engineering [2,3,4]. Microalgae also produce pharmacologically active molecules with immunomodulatory, anti-inflammatory, antihypercholesterolemic, antioxidant, anticancer and antidiabetic properties [5,6,7,8,9,10]. In addition, microalgae are interesting resources in biotechnology, as they can rapidly achieve high levels of biomass production and produce a large quantity of fatty acids (FAs), including monounsaturated FAs (MUFAs), polyunsaturated FAs (PUFAs), and fatty acid methyl esters (FAMEs), which are extremely valuable for various commercial applications. Sami et al. [11] have emphasized the use of algae as a source of antiviral molecules. The chemodiversity of algae and cyanobacteria can be considered as a relevant source for the development of antiviral therapies, as they possess both virus-suppressing properties and immunity-improving capacity [12,13,14]. Hayashi et al. demonstrated that the monogalactosyl diacylglyceride, derived from the green alga *Coccomyxa* sp. KJ, acted as a potent antiviral agent against clinical isolates of severe acute respiratory syndrome coronavirus 2 (SARS-CoV-2) and also against Herpes simplex virus type 1 (HSV-1), both in vitro and in vivo [15,16]. The most known antiviral compounds produced by microalgae include lectins, polysaccharides, pigments, phycobiliproteins, polyphenols (especially flavonoids), peptides, and proteins. Each of them adopts different strategies to fight viruses by interfering with one of the steps of the replication cycle (i.e., adsorption, entry, transcription, capsid assembly) [17]. One of the main mechanisms of action is the direct interaction with the viral envelope, which results in the prevention of the viral entry into the cells inhibiting viral replication. For instance, lectins can directly interact with the high-glycan structure of viral envelope glycoproteins and exhibit anti-HIV activity [18]. Cyanovirin-N is a type of lectin extracted from *Nostoc ellipsosporum*, which has displayed activity against HIV1-, HSV-1, HCV, IAV, and IVB [19]. Polysaccharides generally bind the sites for virus attachment by interacting with the positively charged domains of the virus glycoprotein envelope. Pigments, such as pheophorbide a (PPBa), phycobiliproteins, and carotenoids (e.g., astaxanthin), are extensively used in the biomedical field. PPBa acts both on viral receptors and in post-entry steps [20]. *Spirulina platensis* extracts are rich in allophycocyanin (APC), which can delay the RNA synthesis in vitro of Enterovirus 71 (EV71), a single-stranded RNA virus of the *Piconaviridae* family, causing neurological and cardiovascular disorders [21]. Peptides such as ichthyopeptin A produced by *Microcystis ichthyoblabe* displayed antiviral activity against human influenza virus (IAV) by inhibiting the proteins of the virus cycle [22]. Additionally, some polyphenols affect HCV replication by reducing the ATPase activity and RNA helicase [23]. Considering the literature data, the vast unexploited ecological biodiversity of microalgae holds great promise for the discovery of novel natural products that could find application in the pharmaceutical field and replace the synthetically produced counterparts.

*Galdieria sulphuraria* (Galdieri) Merola (Cyanidiophyceae, Rhodophyta), an ancient extremophilic unicellular red microalga, is able of growing photoautotrophically, heterotrophically, and mixotrophically. Different growing conditions could lead to morphological and biochemical changes with effects on the production of various biomolecules. The heterotrophic growth of *Galdieria sulphuraria* (*G. sulphuraria*) leads to cytological changes in cell size, probably due to the reduced chloroplast size and increased number of mitochondria, which are the organelles directly connected with nutrition [24]. *G. sulphuraria* exhibits high metabolic flexibility that is matched by few other microorganisms, demonstrating its ability to thrive on more than 50 different carbon sources such as sugars, sugar alcohols, tricarboxylic acid cycle intermediates, and amino acids [25]. In addition, *G. sulphuraria* has very high daily productivity rates of various bioactive compounds and significant potential as a source of antioxidants and macronutrients, features that have driven interest in conducting investigations on this species for its potential biotechnological applications [26]. In this study, we analyzed the potential of *G. sulphuraria* as source of antiviral molecules. To test this hypothesis, we evaluated the virucidal effect of its extract against enveloped and non-enveloped viruses and identified the principal classes of metabolites using ultra-high-performance chromatography coupled to high-resolution mass spectrometry (UHPLC-HR MS).

## 2. Results

### 2.1. Chemical Characterization

The untargeted approach disclosed the presence of 11 compounds—mainly lipids, as well as two chlorophyll derivatives. In Table 1, the UHPLC-HRMS data used for tentative identification are provided.

In electrospray negative ion mode, palmitic acid (16:0) was the only detected saturated free fatty acid, which was also found in its amide form (C_16_H_33_NO; *m*/*z* 256.2629, mass error −2.3 ppm) as a protonated adduct. Five other compounds structurally related to this were tentatively identified, all of which derived from 18-carbon-chain fatty acids, both saturated and unsaturated (Figure S1). In particular, the compound with the molecular formula C_18_H_33_NO (*m*/*z* 280.2631, RDB 3) was tentatively identified as octadecadienamide (18:2), likely linoleamide, together with its hydroxyl derivative (C_18_H_33_NO_2_) [27]. Two isomers of octadecenamide (e.g., oleamide; 18:1) showed a deprotonated molecular ion at *m*/*z* 282.2791 (theoretical value), whereas the ion at *m*/*z* 284.2939 (mass error −3.1) was putatively associated with stearamide (18:0). Lipid amides accounted for 88% of the tentatively identified compounds, whereas pigments were minor constituents, representing 2%, based on peak areas (Figure S2). These latter ones, eluting at 23.308 and 30.259, were tentatively identified as pheophorbide a and hydroxypheophytin a, respectively. Apart from molecular formulas, in line with the presence of chlorin rings containing four nitrogen atoms, further confirmation was found in the HR-MS/MS data (Figure S3) compared with the literature [28]. The phytol moiety in the structure of hydroxypheophytin a was evidenced by the loss of 278 Da from the protonated molecular ion to provide the fragment ion at *m*/*z* 609.2711.

### 2.2. Evaluation of Cytotoxicity Effects

**Figure 1:**
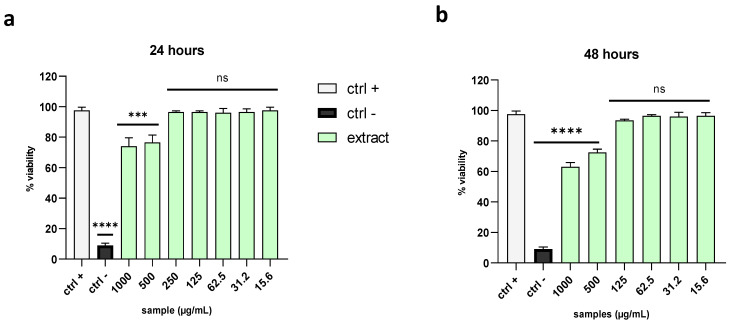
Cytotoxicity evaluation. The viability of Vero cells was analyzed after treatment with the extract at (**a**) 24 h and (**b**) 48 h. Dimethyl sulfoxide (DMSO) was used as the negative control (ctrl−), while non-treated cells were used as the positive control (ctrl+). Note: **** *p* < 0.0001; *** *p* = 0.0001; ns: non-significant.

Before exploring the inhibitory potential of the algal extract against a broad range of human viruses, we assessed its effect on cell viability. Therefore, the MTT assay was performed by exposing Vero cells to different extract concentrations ranging from 15.6 to 1000 µg/mL. The cells were treated with the extract for 24 and 48 h, after which the absorbance of solubilized formazan crystals was measured. By setting a threshold line at the 50% cytotoxic concentration (CC_50_), the extract did not show any relevant cytotoxicity at 24 h (Figure 1a) or 48 h (Figure 1b) after treatment.

### 2.3. Evaluation of Antiviral Activity on Different Viruses

To evaluate the antiviral activity of the algal extract and its potential mechanism of action, we performed the plaque assay under four different treatment conditions (explained in the Section 4) against human viruses with different genomes (DNA or RNA) and envelopes (with or without).

HSV-1 and HSV-2 were used as models of enveloped DNA viruses, while HCoV-229E and SARS-CoV-2 were used as models of enveloped RNA viruses. Finally, poliovirus 1 (PV-1) was employed as a non-enveloped virus.

#### 2.3.1. Enveloped Viruses

**Figure 2:**
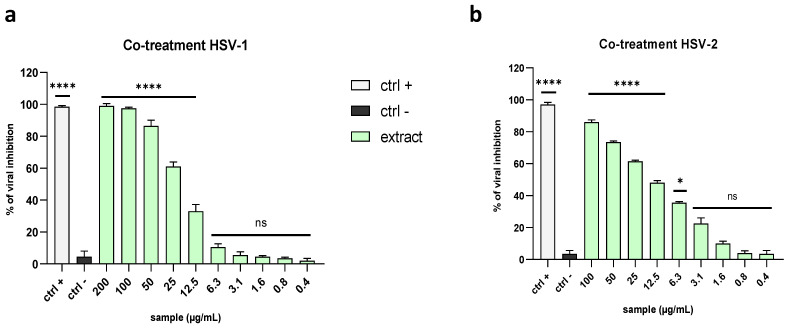
Comparison of antiviral activity levels against Herpesviridae members in a co-treatment assay. The antiviral potential of the extract was evaluated against (**a**) HSV-1 and (**b**) HSV-2. The extract was able to block the viral replication of both viruses. Rhamnolipid M15RL at 50 µg/mL [31] was used as the positive control (ctrl+), while infected and untreated cells were used as the negative control (ctrl−). Note: **** *p* < 0.0001; * *p* = 0.0211; ns: non-significant.

Since envelope proteins of many viruses allow viral entry into host cells, we wanted to verify whether the algal extract could interfere with the interaction between the cell surface and envelope glycoproteins, thereby preventing binding, fusion with the host membrane, and viral replication [29]. First, we assessed the activity against two members of the Herpesviridae family, namely HSV-1 and HSV-2. This group involves linear double-stranded DNA viruses, whose genome is enclosed in an icosahedral capsid and an external lipid bilayer known as the envelope [30]. Initially, we performed a co-treatment assay to verify whether the simultaneous presence of the virus and extract on cells could affect the infection. Then, 48 h post-infection (p.i.), the mixture was removed and replaced with a dense medium consisting of carboxymethyl cellulose (CMC) diluted in the culture medium. The results demonstrated that the extract exhibited relevant antiviral activity against the Herpesviridae family, showing 50% inhibition at 20.7 µg/mL against HSV-1 and 12.4 µg/mL against HSV-2 (Figure 2a,b).

**Figure 3:**
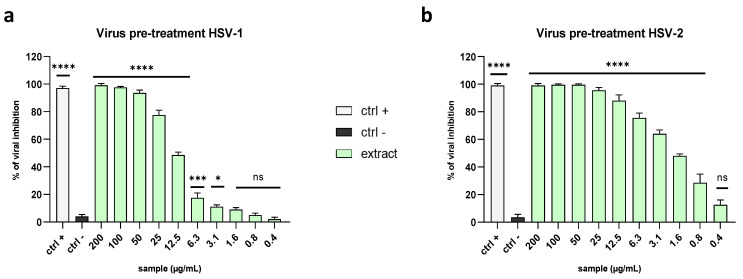
Comparison of antiviral activity levels against Herpesviridae members in the virus pre-treatment assay. The raw extract inhibited the early stages of the infection both against (**a**) HSV-1 and (**b**) HSV-2, exhibiting higher antiviral potency against the latter. Rhamnolipid M15RL at 50 µg/mL [31] was used as a positive control (ctrl+), while infected and untreated cells were used as the negative control (ctrl−). Note: **** *p* < 0.0001; *** *p* = 0.0002; * *p* = 0.0319; ns: non-significant.

To investigate in which stage of the viral infection (extracellular or intracellular) the extract could act, assays with different experimental procedures were performed. The virus pre-treatment assay was carried out by allowing the virus (0.1 MOI) and the extract to interact for 1 h, then the mixture was used to infect the cell monolayer. The extract exhibited a more potent effect against HSV-1 and HSV-2 than the related co-treatment assay, with half-maximal inhibitory concentration (IC_50_) values of 12.3 µg/mL and 1.7 µg/mL, respectively (Figure 3a,b). These data are very promising, indicating that the extract could interact directly with the viral particles, perhaps by binding with some envelope glycoproteins and blocking viral entry.

**Figure 4:**
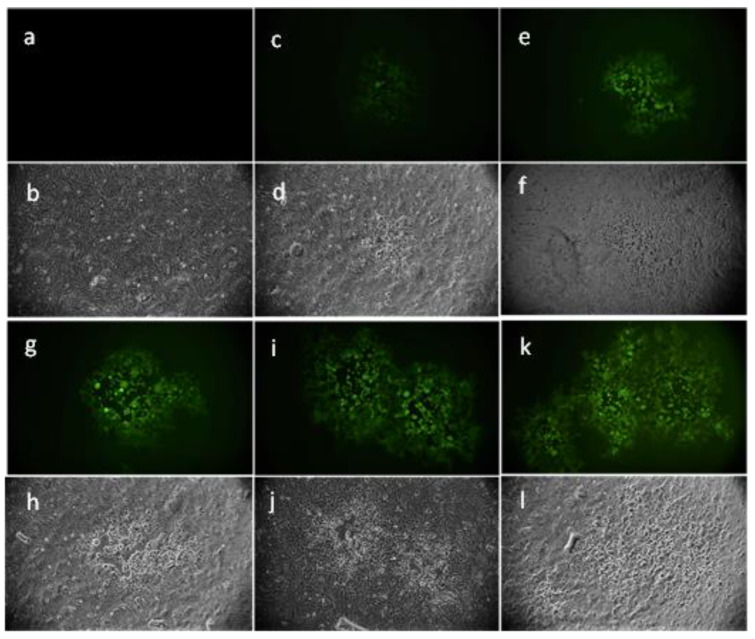
Fluorescence microscopy images of cells infected with HSV-1-GFP in a virus pre-treatment assay. Images were acquired after 48 h of infection using (**a**,**c**,**e**,**g**,**i**,**k**) fluorescein isothiocyanate (FITC) and (**b**,**d**,**f**,**h**,**j**,**l**) bright field (BF) filters. No plaques were detected at the highest concentration of 100 µg/mL (**a**,**b**). The size and amount of the plaques increased proportionally with the decreased extract concentrations. The antiviral effects at (**c**,**d**) 50 µg/mL, (**e**,**f**) 25 µg/mL, (**g**,**h**) 12.5 µg/mL, and (**i**,**j**) 6.3 µg/mL (**k**,**l**) were observed. Negative control (ctrl−) shows untreated and infected cells.

The inhibition of HSV-1 in the virus pre-treatment was also confirmed by tracking the fluorescence through an inverted fluorescence microscope. Briefly, the virus pre-treatment was performed by using the engineered HSV-1, which incorporated GFP in the gene encoding VP22 tegument protein (Figure 4). The acquired images showed a total absence of fluorescence at 100 µg/mL (Figure 4a,b), indicating complete inhibition, in accordance with the relative plaque assay (100% inhibition). Then, proportional increases in the amount, shape amount, and fluorescence intensity of the viral plaques were observed at the lower concentrations (Figure 4c–j) until reaching the same number of plaques of the negative control, at 6.3 µg/mL of algal extract, causing an intense fluorescence signal (Figure 4i,j). These data were in line with the plaque assay experiment.

**Figure 5:**
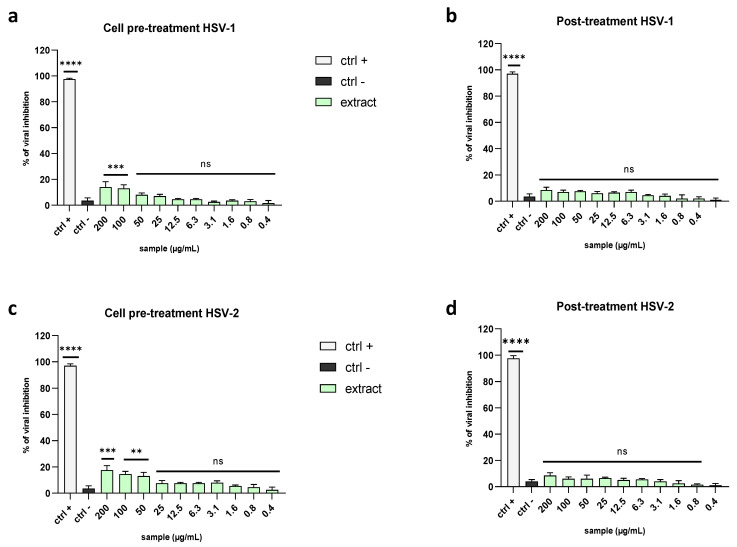
Comparison of antiviral activity levels against Herpesviridae members in cell pre-treatment and post-treatment assays. The antiviral potential of the extract was evaluated in (**a**) cell pre-treatment and (**b**) post-treatment assays against HSV-1 and (**c**) cell pre-treatment and (**d**) post-treatment assays against HSV-2. Two positive controls were used: (**b**) dextran sulfate (1 µM) in the cell pre-treatment and (**a**) aciclovir (5 µM) in the post-treatment. Infected and untreated cells were used as the negative control (ctrl−). Note: **** *p* < 0.0001; *** *p* = 0.0002; ** *p* = 0.001; ns: non-significant.

To clarify whether the algal extract could also interact with the host cells by creating a protective barrier on the cell surface and preventing the binding between viral glycoproteins and cell membrane receptors, we performed a cell pre-treatment assay. Therefore, Vero cells were pre-treated with the extract before viral infection occurred. The algal extract did not display any significant effect (Figure 5a,c), suggesting that it did not interfere with the cell surface to stop the infection.

The same negative results were obtained in the post-treatment assay, where first the cell monolayer was infected by the virus and then the viral suspension was replaced with the extract dilutions. This type of treatment allowed us to assess the ability of the extract to affect viral replication after intracellular entry. The data confirmed that the algal extract could not block the virus’ replication once inside cells (Figure 5b,d).

Even though herpesviruses can cause severe diseases, in general, DNA viruses are not associated with severe pandemics caused by RNA viruses, such as Zika, avian influenza, West Nile, Ebola, Chikungunya, and other viruses [32]. Therefore, to assess the antiviral activity of the algal extract against RNA viruses, we performed all of the described treatments against members of the *Coronaviridae* family, namely HCoV-229E and SARS-CoV-2, the causative agent of the current pandemic.

**Figure 6:**
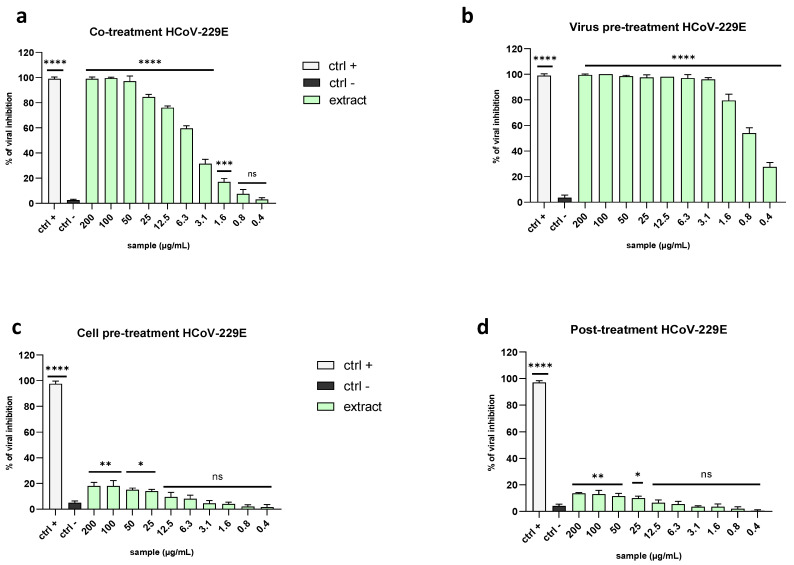
Antiviral activity against the alphacoronavirus HCoV-229E. Different assays were performed to explore the antiviral power of the extract against HCoV-229E: (**a**) co-treatment; (**b**) virus pre-treatment; (**c**) cell pre-treatment; (**d**) post-treatment. The extract exhibited a stronger antiviral effect in the virus pre-treatment assay. The positive controls (ctrl+) are reported below: (**a**,**b**) rhamnolipid M15RL at 50 µg/mL [31] was used in the co-treatment and virus pre-treatment, (**c**) ivermectin (10 µM) in the cell pre-treatment, and (**d**) remdesivir (10 µM) in the post-treatment. Infected and untreated cells were used as the negative control (ctrl−). Note: **** *p* < 0.0001; *** *p* = 0.0002; ** *p* = 0.001; * *p* = 0.03; ns: non-significant.

As shown in Figure 6, the extract was far more potent against coronaviruses than herpesviruses. In detail, it exhibited 50% inhibition against HCoV-229E in the co-treatment assay at 5.3 µg/mL (Figure 6a) and a 4-fold reduction in the viral titer at the same concentration in the virus pre-treatment assay, with an IC_90_ at 1.8 µg/mL (Figure 6b).

In both the cell pre-treatment and post-treatment assays, the plaque analysis did not reveal any signs of viral inhibition (Figure 6c,d).

**Figure 7:**
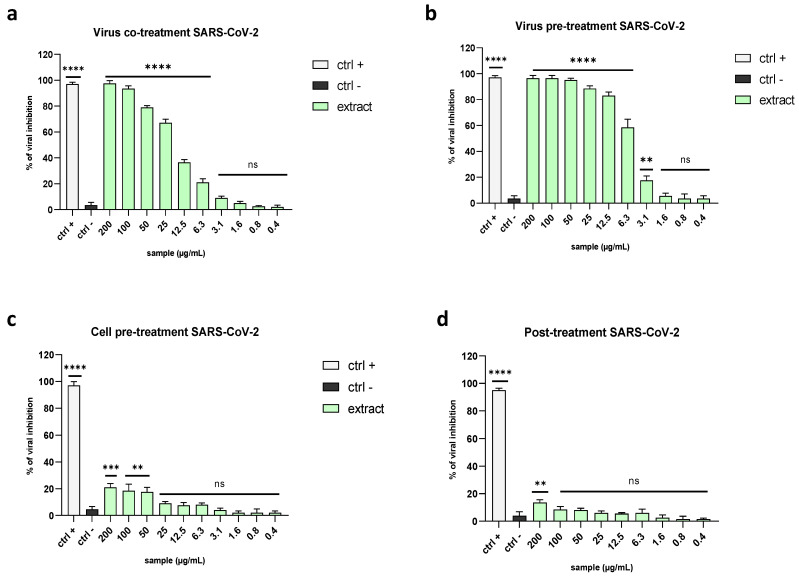
Antiviral activity levels against the betacoronavirus SARS-CoV-2. Different assays were performed to explore the antiviral power of the extract against SARS-CoV-2: (**a**) co-treatment: (**b**) virus pre-treatment; (**c**) cell pre-treatment; (**d**) post-treatment. The data indicated that the extract inhibited viral replication by preventing the entry into the cells. The positive controls (ctrl+) are reported below: (**a**,**b**) rhamnolipid M15RL at 50 µg/mL [31] was used in the co-treatment and virus pre-treatment, (**c**) ivermectin (10 µM) in the cell pre-treatment, and (**d**) remdesivir (10 µM) in the post-treatment. Infected and untreated cells were used as negative controls (ctrl−). Note: **** *p* < 0.0001; *** *p* = 0.0003; ** *p* = 0.002; ns: non-significant.

The great results against HCoV-229E prompted us to test the algal extract against the causative agent of the current pandemic, namely SARS-CoV-2. The results are in line with the previous data, showing the inhibition of viral entry. Indeed, the extract induced a 67% decrease in viral replication when tested at 25 µg/mL in a co-treatment assay and a 58% decrease at 6.3 µg/mL in the virus pre-treatment (Figure 7a,b), with IC_50_ values of 16.9 and 5.7 µg/mL, respectively. In the same way, there was no activity in the other treatments (Figure 7c,d).

All these data demonstrated that the algal extract interacts with some component present in the viral envelope, preventing viral replication upstream, thereby inhibiting the activity against HSV-2 and HCoV-229E.

#### 2.3.2. Non-Enveloped Viruses

To confirm that the algal extract could target the viral envelope, we tested it against a non-enveloped virus belonging to the Picornaviridae family, the poliovirus type 1 (PV-1). It is a positive-strand RNA virus whose genetic material is contained only in a protein capsid shell.

**Figure 8:**
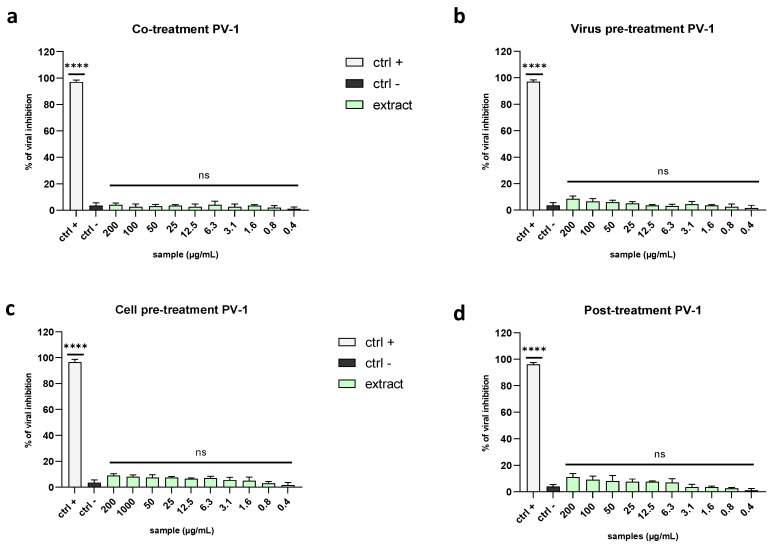
Antiviral activity against a non-enveloped virus. Different assays were performed to explore the antiviral power of the extract against PV-1: (**a**) co-treatment; (**b**) virus pre-treatment; (**c**) cell pre-treatment; (**d**) post-treatment. The data showed that the extract did not inhibit viral replication in any treatment. Positive controls (ctrl+) are reported below: (**a**,**b**) pleconaril (2 µg/mL) for the co-treatment and virus pre-treatment, (**c**) WIN51711 (5 µg/mL) for the cell pre-treatment, and (**d**) protein 2C (10 µM) for the post-treatment. Infected and untreated cells were used as the negative control (ctrl−). Note: **** *p* < 0.0001; ns: non-significant.

The obtained data suggested that the algal extract could block the spread of enveloped viruses but not that of the non-enveloped ones, probably because it interacted with the envelope during the early stages of infection, thereby preventing the virus’ entry (Figure 8a–d).

### 2.4. Evaluation of Viral Gene Expression

To further investigate the effect of the algal extract, we quantified its inhibitory activity through a quantitative RT-PCR. We evaluated the relative expression of the following genes: immediate-early (IE) gene UL54, early (E) gene UL52, and late (L) gene UL27 for HSV-2; spike gene (S) and RNA-dependent RNA polymerase (RdRp) protein for HCoV-229E; spike gene (S) and nucleocapsid gene (N) for SARS-CoV-2. In detail, UL54 is the first of the three selected genes to be encoded when viral entry occurs. It encodes the infected cell culture polypeptide 27 (ICP27) protein, which is essential for the viral infection of cells. It regulates transcription, splicing events, and nuclear export, and plays an essential role in the expression of late viral genes [33]. The early gene UL52 encodes the DNA primase, a key factor required for the beginning of viral DNA replication [34]. The UL27 gene encodes the late envelope glycoprotein B (gB), a class III fusion protein, which drives the onset of infection by allowing the fusion of virus and host cell membranes [35].

**Figure 9:**
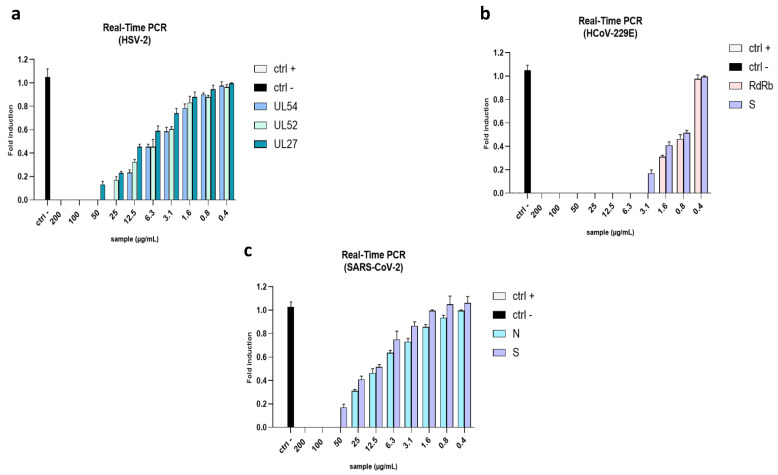
Quantitative RT-PCR. The molecular assay was performed to evaluate the role of the algal extract in the reduction in viral gene expression after a virus pre-treatment assay. (**a**) The expression of UL54, UL52, and UL27 in HSV-2. (**b**) The expression of RdRb and S proteins in HCoV-229E. (**c**) The expression of N and S in SARS-CoV-2. Ctrl− refers to infected but untreated cells.

As scheduled, in the virus pre-treatment, no detectable levels of the genes were recorded at the highest concentrations, while reduced expression levels were found at a lower concentration (Figure 9a). The expression levels of S and N genes were analyzed to confirm the antiviral effect on HCoV-229E and SARS-CoV-2. The S gene encodes the spike protein, an essential factor in viral entry; the nucleocapsid protein, encoded by the N gene, is crucial in the viral assembly, replication, and regulation of the host immune response [36,37,38,39]. Similar to herpes genes, the graph in Figure 9b indicates that the extract determined a dose-dependent reduction in the expression of coronaviruses genes.

## 3. Discussion

The current SARS-CoV-2 pandemic has highlighted the importance of searching for new antivirals as quickly as possible to face the onset of new variants and to counteract new pandemic events. The solution could be found in some still unexplored ecosystems. Several aquatic microorganisms, for example, are able to produce biologically active secondary metabolites under stressful conditions, such as low-pH, high-salinity, and high-temperature conditions, which could have important applications in human life. These natural compounds represent a viable alternative to synthetic pharmaceutical products whose production is often expensive, polluting, and energy-consuming. For all these reasons the present study focused on the search for antiviral compounds derived from the red microalga *G. sulphuraria*, an extremophilic alga isolated from a hot sulfur spring. This species is well-studied for its potential role in bioremediation and biofuel production. Selvaratnam et al. demonstrated that *G. sulphuraria* was able to remove nutrients from primary wastewater effluent, such as phosphates and ammoniacal nitrogen, and sugars from fruit salad production [40]. Other studies reported that this alga could also be used in metal removal, such as for gold, rare elements, palladium, and negative charged platinum (PtCl_6_^2−^). Moreover, it is a good candidate for producing bio-oils through hydrothermal liquefaction [41,42,43,44,45]. Nevertheless, nothing has been reported on its antiviral properties. In our study, the freeze–thaw method was applied to extract the red microalga following conventional extraction using acetone as the extracting solvent. Fatty acid amides were unraveled as the main constituents. Liu et al. recently reported on oleamide in *Galdieria sulphuraria* grown under both mixotrophic and autotrophic culture conditions [46]. Indeed, the first chlorophyll derivative was previously characterized in the microalga cultured under acidic conditions [47]. We obtained a mixture enriched mostly in primary fatty acid amides (PFAM), i.e., linoleamide, oleamide, palmitamide, stearamide, and hydroxylinoleamide, which represent bioactive signaling lipids found in animals, grasses, and microalgae [48]. Among them, oleamide is related to a wide range of activities; for example, it is a very common slip agent used in industrial applications. In addition, oleamide is a potential antifoulant coating material and could be used to avoid the initial biofilm formation and diatom attachment [48]. We hypothesized that the strong antiviral effect of our extract could be ascribed to its surfactant properties. Indeed, it is known that surfactants inhibit viral replication by interfering with biological membranes [31,49]. Another key component identified by UHPLC-HR MS is PPBa, a chlorophyll derivative with reported antiproliferative effects on several tumoral cell lines [48]. Recently, Meunier et al. discovered that PPBa exerted strong antiviral activity against a broad range of enveloped viruses, including SARS-CoV-2, directly targeting viral particles [50,51]. Our mixture of fatty acid amides exhibited very potent antiviral activity against DNA and RNA viruses whose common feature is the presence of the envelope, while it did not act against naked viruses. Fatty acids, which are structurally related to the identified amides, are well-known for their antimicrobial activity due to their capability to induce the complete disruption of microbial cell membranes and viral envelopes. The antimicrobial power increases with the increase in their unsaturation levels. Our results demonstrated that the algal extract exhibited a strong antiviral effect against the members of the *Herpesviridae* (HSV-1 and HSV-2) and *Coronaviridae* (HCoV-229E and SARS-CoV-2) families when it was pre-incubated with viruses. This means it was able to interact with envelope glycoproteins to prevent cell entry. HSV-1 and -2 are commonly associated with recurrent oral and genital lesions. Their envelope is composed of 12 glycoproteins that are involved in HSV cell entry, virus egress, and cell-to-cell spread [52]. Although HSV-1 and HSV-2 share similar features (i.e., they share >80% amino acid similarity), the extract displayed different inhibition efficiency rates between the two species (i.e., IC_50_ at 12.5 µg/mL for HSV-1 and 6.1 µg/mL for HSV-2), which could be due to the distinct abilities of glycoproteins to provide unspecific electrostatic bonds [53,54,55]. In addition, it is known that the two species evolved under different evolutionary pressures, as demonstrated by Lames et al., who showed a distinct arrangement of envelope glycoproteins resulting in different viral spread rates, with HSV-1 providing higher yields than HSV-2 [56]. The same activity was recorded against *Coronaviridae* members, with IC_50_ values of 6.3 µg/mL for SARS-CoV-2 and 0.8 µg/mL for HCoV-229E, suggesting that the extract was able to block viral infection by also interacting with the spike fusion glycoprotein. These promising results, supported by the molecular analysis (RT-qPCR), demonstrated for the first time that *G. sulphuraria* is a producer of antiviral compounds able to drastically interfere with HSV-1 and coronavirus life cycles.

## 4. Materials and Methods

### 4.1. Galdiera Sulphuraria Cultivation

*G. sulphuraria* (strain 064) was obtained from the Algal Collection of University Federico II (ACUF), University of Naples, Italy. The strain was maintained in 100 mL Erlenmeyer flasks containing Allen medium supplemented with (NH_4_)_2_SO_4_ as the nitrogen source (Sigma-Aldrich, Darmstadt, Germany), at 37 °C and pH 1.8. The flasks were continuously stirred and illuminated under a 24/24 regimen from below with a warm-white LED at a photon flux density of 400 μmol m^−2^ s^−1^. The algae were grown in autotrophic conditions in Allen medium ((NH_4_)_2_SO_4_ 1320 mg/L, MgSO_4_ × 7H_2_O 300 mg/L, K_2_HPO_4_ 300 mg/L, KH_2_PO_4_ 300 mg/L, CaCl_2_ × 2H_2_O 20 g/L, NaCl 200 mg/L, FeSO_4_ × 7H_2_O 996 mg/L) with 10 mL of trace elements 1× (MnCl_2_ × 4H_2_O 2 mg/L, H_3_BO_3_ 21 mg/L, CuSO_4_ × 5H_2_O 1 mg/L, Na_2_MoO_4_ × 2H_2_O 1 mg/L, CoCl_2_ × H_2_O 1 mg/L, ZnCl_2_ 1 mg/L). The medium was acidified by adding H_2_SO_4_. For the mass cultures, inocula from flasks were transferred to continuously stirred 1 L bubble column photobioreactors at 37 °C and pH 1.8 under the same light conditions. To ensure uniform exposure to light and increased productivity, a mixture of air and 2% CO_2_ was injected into the system continuously. The algal growth was monitored both by measuring the optical density (OD) at 750 nm and counting cells/mL in a Bürker chamber.

### 4.2. Extract Preparation

The algae were collected during the exponential growth phase and subsequently extracted using a freeze–thaw method followed by extraction with acetone [57]. Then, the cells were collected by centrifugation at 6000 rpm for 15 min and the pellet was dissolved in 20 mL of distilled water and subjected to heat shock. Briefly, the cells were exposed for a short time to temperatures above and below the normal growth conditions (80 °C and −80 °C, respectively). The solution was overlayed with five times the volume of 95% ethanol and incubated overnight at −20 °C. After the collection, the pellet was subjected to organic extraction twice with 10 mL of acetone. The supernatant was recovered by centrifugation and evaporated using a rotavapor (R-100, BUCHI, Flawil, Switzerland). The dried extract was weighted, partly (1 mg) stored dissolved in 100% dimethyl sulfoxide (DMSO, Sigma-Aldrich) at the concentration of 10 mg/mL, and the stored at −20 °C until a cytotoxicity and antiviral screening was carried out; 1 mg of the dried extract was stored at −20 °C until the UHPLC HR-MS analysis was performed. For this purpose, the sample was dissolved in methanol (1 mL; LC-MS Ultra CHROMASOLV™, Honeywell Riedel-de Haën™).

### 4.3. Ultra-High Performance Liquid Chromatography–High-Resolution Mass Spectrometry Analyses

The UHPLC method was carried out on a NEXERA UHPLC system (Shimadzu, Tokyo, Japan) using a Luna^®^ Omega C18 column (50 × 2.1 mm i.d., 1.6 μm particle size; Phenomenex, Torrance, CA, USA). The mobile phase consisted of H_2_O and CH_3_CN, both acidified with HCOOH (0.1%). A linear elution gradient was started from 30% B, held for 1 min, then ramped to 95% B in 29 min. Then, it was kept for 1 min before restoring the initial conditions. The flow rate was 0.5 mL/min and the injection volume was 2 µL. HR MS analyses were performed by using the AB SCIEX TripleTOF^®^ 4600 spectrometer (AB Sciex, Concord, ON, Canada), equipped with a DuoSpray™ ion source operating in both negative and positive electrospray (ESI) ion modes. The APCI probe was used for automated mass calibrations of all scan functions using the Calibrant Delivery System. An untargeted approach was developed, consisting of a full-scan TOF survey in the mass range of 100–1500 Da with an accumulation time of 250 ms, as well as eight information-dependent acquisition MS/MS scans in the mass range of 80–1300 Da with an accumulation time of 100 ms. The applied source parameters were the following: curtain gas (CUR) at 35 psi, nebulizer gas (GS 1) at 60 psi, heated gas (GS 2) at 60 psi, an ion spray voltage (ISVF) of +5.5 (−4.5) kV, an interface heater temperature of 600 °C, a declustering potential of −80 V, and a collision energy of 45 V with a spread of 15 V. The instrument was controlled by Analyst^®^ TF 1.7 software, and data processing was performed using PeakView^®^ software version 2.2.

### 4.4. Cell Lines and Viral Strains

African green monkey kidney epithelial cell lines (Vero CCL-81, Manassas, VA, USA) were acquired from the American Type Culture Collection (ATCC), cultivated in Dulbecco’s modified Eagle’s medium (DMEM) with 4.5 g/L glucose (Microtech, Naples, Italy) supplemented with 10% heat-inactivated fetal bovine serum (FBS, Microgem, Naples, Italy), 2 mM L-glutamine (Microtech), and 100 IU/mL of penicillin–streptomycin solution (Himedia, Naples, Italy), and maintained at 37 °C in a humidified atmosphere with 5% CO_2_. All described viral strains were propagated on Vero cell lines: HSV-1 (strain SC16), HSV-1expressing the VP22-green fluorescent protein, HSV-2 (strain 333), poliovirus type 1 (PV-1 strain CHAT), human coronavirus 229E (HCoV-229E strain VR-740), and SARS-CoV-2 (strain VR-PV10734; kindly provided by Lazzaro Spallanzani Hospital, Rome, Italy) [58]. The experiments involving SARS-CoV-2 were carried out in a biosafety level 3 (BSL-3) containment laboratory (University of Salerno) according to World Health Organization (WHO) recommendations.

### 4.5. Cytotoxicity Assay

The effect of the raw extract on the cell viability was evaluated using a 2,5-diphenyl-2H-tetrazolium bromide (MTT) assay. Vero cells were seeded into a 96-well cell culture plate at an initial concentration of 2 × 10^4^ cells for each well. After 24 h of incubation, the cells were treated with serial dilutions of the raw extract (500, 250, 125, 62.5, 31.2, 15.6 µg/mL) for 24 and 48 h. Cells without compounds were used as the negative control. After the treatment, the cells were left to react with the MTT for about 3 h in the dark. The formazan crystals produced by the viable cells were solubilized in DMSO after removing the supernatant, and the absorbance was measured at 570 nm with a TECAN M-200 reader (Tecan, Männedorf, Switzerland). The cell survival was calculated by the following formula:

(OD treated well [−blank])/(mean OD control well [−blank]) × 100

### 4.6. Antiviral Activity Assay

The potential antiviral activity of the extract was evaluated through the plaque assay. Basically, Vero cells were seeded in 24-well plates at the initial concentration of 1.3 × 10^5^ cells/well and incubated overnight. The monolayer was treated with different extract concentrations, ranging from 200 µg/mL to 1 µg/mL, and infected with viruses at 0.01 multiplicity of infection (MOI). To understand in which stage of the infection the extract could act, the assay was performed in four different experimental conditions: a co-treatment assay, in which the monolayer was treated and infected simultaneously with the extract and virus and incubated for 1 h (adsorption time); a virus pre-treatment assay, in which the virus and extract were incubated together for 1 h at 0.1 MOI. The solution was then diluted and used to infect the monolayer at 0.01 MOI; for the cell pre-treatment, in which the cell monolayer was first treated with the extract for 1 h and then infected with the virus; and for the post-treatment assay, in which the cell monolayer was first infected with the virus and then treated with the extract for 1 h. For each treatment, after the viral adsorption time, the monolayer was washed with citrate buffer (pH 3) to remove the non-penetrated viral particles. Then, it was covered with DMEM supplemented with 3% carboxymethyl cellulose (CMC) and finally incubated for 24–72 h. At the end of the treatment, the cells were fixed with 4% formaldehyde and stained with 0.5% crystal violet.

The percentage of viral inhibition was calculated by counting the number of plaques compared to the infected and non-treated cells (ctr−) as follows:

% viral inhibition = (1 − (number of plaques in treated cells)/(number of plaques in negative control)) × 100

### 4.7. Fluorescence Microscopy

The virus pre-treatment assay was performed by using HSV-1 expressing the VP22-GFPPMID construct. At 48 h post-infection, the fluorescence was recorded and images were acquired through the Nikon ECLIPSE Ti2-U (Nikon Europe B.V., Amsterdam, The Netherlands) inverted fluorescence microscope.

### 4.8. Gene Expression Analysis: Real-Time PCR

The virus pre-treatment was performed as described above. After 24 h of infection, the total RNA was extracted by the TRIzoL reagent (Thermo Fisher, Waltham, MA, USA) [59]. Next, 1 µg of RNA was retrotranscribed with a SensiFAST™ cDNA Synthesis Kit (Meridian Bioscience, Washington, DC, USA) to cDNA. Real-Time PCR (RT-PCR) tests were run in triplicate, whereby 2 µL of cDNA was amplified using 2× SensiFAST™ SYBR^®^ No-ROX Mix (Meridian Bioscience, Washington, DC, USA) and 0.4 µM of primers, at a final volume of 20 µL. The relative target threshold cycle (Ct) values of UL52 and UL27 (for HSV-2), spike (S) protein and RNA-dependent RNA polymerase (RdRp) protein (for HCoV-229E), and spike (S) protein and nucleocapsid (N) protein (SARS-CoV-2) were normalized to glyceraldehyde 3-phosphate dehydrogenase (GAPDH) as the housekeeping gene. The primers are listed in Table 2. The relative expression levels of the genes of interest were expressed using the 2^−∆∆Ct^ method.

### 4.9. Statistical Analysis

Each test was performed in triplicate and expressed as the mean ± standard Deviation (SD) calculated by GraphPad Prism (version 5). Statistical differences were evaluated via a one-way ANOVA followed by a Dunnett test; a value of *p* ≤ 0.05 was considered significant.

## 5. Conclusions

*G. sulphuraria* is considered a promising organism thanks to its extremophilic properties. In our study, we assessed that the extract was able to produce important compounds with antiviral properties. In detail, this mixture of compounds exhibited significant antiviral activity against members of the *Herpesviridae* and *Coronaviridae* families at no cytotoxic concentrations. In addition, our results suggested that the mixture could block the infection of enveloped viruses by interacting with important surface components, leading to significant damage to the viral envelope. These promising results pave the way for the further purification of the compounds and investigations into its mode of action for its potential application as an antiviral agent in the pharmaceutical field.

## Figures and Tables

**Table 1:** UHPLC-HRMS data (Rt = retention time; (−) (+) = ESI ion mode; RDB = ring and double bond value).

Rt (min)	Tentative Identification	Molecular Formula	*m*/*z* Found	*m*/*z* Calc.	Error (ppm)	RDB
17.934	Octadecadienamide	C_18_H_33_NO	280.2631 (+)	280.2635	−1.4	3
18.501	Hydroxyoctadecadienamide	C_18_H_33_NO_2_	296.2579 (+)	296.2584	−1.7	3
22.076	Palmitamide	C_16_H_33_NO	256.2629 (+)	256.2635	−2.3	1
22.500	Octadecenamide 1	C_18_H_35_NO	282.2783 (+)	282.2791	−2.8	2
22.831	Octadecenamide 2	C_18_H_35_NO	282.2785 (+)	282.2791	−2.3	2
23.052	Palmitic acid	C_16_H_32_O_2_	255.2337 (−)	255.2330	+2.9	1
23.308	Pheophorbide a	C_35_H_36_N_4_O_5_	593.2731 (+)	593.2758	−4.6	20
24.200	Stearamide	C_18_H_37_NO	284.2939 (+)	284.2948	−3.1	1
29.638	Docosatetraenoylethanolamine	C_25_H_43_NO_2_	390.3349 (+)	390.3367	−4.5	5
29.646	Tetracosanoylethanolamine	C_26_H_45_NO_2_	404.3507 (+)	404.3523	−4.0	5
30.259	hydroxypheophytin a	C_55_H_74_N_4_O_6_	887.5637 (+)	887.5681	−4.9	21

**Table 2:** Viral genes and relative primers used for real-time PCR.

Gene	Virus	Forward Sequence	Reverse Sequence
UL54 UL52 UL27	HSV-2	TGGCGGACATTAAGGACATTG GACCGACGGGTGCGTTATT GCCTTCTTCGCCTTTCGC	TGGCCGTCAACTCGCAG GAAGGAGTCGCCATTTAGCC CGCTCGTGCCCTTCTTCTT
S RdRp	HCoV-229E	CGTTGAACTTCAAACCTCAGA	ACCAACATTGGCATAAACAG
S N	SARS-CoV-2	AGGTTGATCACAGGCAGACT GGGGAACTTCTCCTGCTAGAAT	GCTGACTGAGGGAAGGAC CAGACATTTTGCTCTCAAGCTG
GAPDH	/	CCTTTCATTGAGCTCCAT	CGTACATGGGAGCGTC

## Data Availability

The data presented in this study are available on request from the corresponding author. The authors can confirm that all relevant data are included in the article.

## Supplementary Materials

The following supporting information can be downloaded at: https://www.mdpi.com/article/10.3390/md21070383/s1. Figure S1: Extracted ion chromatograms (XICs) of detected and tentatively identified fatty acid amides. Figure S2: TICs (total ion chromatograms) acquired in ESI(−) ion mode (upper panel) and ESI(+) ion mode (lower panel). Relative quantitation data are also reported. Figure S3: TOF-MS/MS spectrum of (A) pheophorbide a and (B) hydroxypheophytin a.

## References

[1] Tompa D.R., Immanuel A., Srikanth S., Kadhirvel S. (2021). Trends and strategies to combat viral infections: A review on FDA approved antiviral drugs. Int. J. Biol. Macromol..

[2] Carbone D.A., Pellone P., Lubritto C., Ciniglia C. (2021). Evaluation of Microalgae Antiviral Activity and Their Bioactive Compounds. Antibiotics.

[3] Kim S.K., Vo T.S., Ngo D.H. (2011). Potential application of marine algae as antiviral agents in medicinal foods. Adv. Food Nutr. Res..

[4] Raihan T., Rabbee M.F., Roy P., Choudhury S., Baek K.H., Azad A.K. (2021). Microbial Metabolites: The Emerging Hotspot of Antiviral Compounds as Potential Candidates to Avert Viral Pandemic Alike COVID-19. Front. Mol. Biosci..

[5] Pham T.X., Lee Y., Bae M., Hu S., Kang H., Kim M.B., Park Y.K., Lee J.Y. (2019). Spirulina supplementation in a mouse model of diet-induced liver fibrosis reduced the pro-inflammatory response of splenocytes. Br. J. Nutr..

[6] Samuels R., Mani U.V., Iyer U.M., Nayak U.S. (2002). Hypocholesterolemic effect of spirulina in patients with hyperlipidemic nephrotic syndrome. J. Med. Food.

[7] Balasubramaniam V., Gunasegavan R.D., Mustar S., Lee J.C., Mohd Noh M.F. (2021). Isolation of Industrial Important Bioactive Compounds from Microalgae. Molecules.

[8] Pulz O., Gross W. (2004). Valuable products from biotechnology of microalgae. Appl. Microbiol. Biotechnol..

[9] Lauritano C., Andersen J.H., Hansen E., Albrigtsen M., Escalera L., Esposito F., Helland K., Hanssen K.Ø., Romano G., Ianora A. (2016). Bioactivity Screening of Microalgae for Antioxidant, Anti-Inflammatory, Anticancer, Anti-Diabetes, and Antibacterial Activities. Front. Mar. Sci..

[10] Ku C.S., Pham T.X., Park Y., Kim B., Shin M.S., Kang I., Lee J. (2013). Edible blue-green algae reduce the production of pro-inflammatory cytokines by inhibiting NF-κB pathway in macrophages and splenocytes. Biochim. Biophys. Acta.

[11] Sami N., Ahmad R., Fatma T. (2021). Exploring algae and cyanobacteria as a promising natural source of antiviral drug against SARS-CoV-2. Biomed. J..

[12] Kasting J.F., Siefert J.L. (2002). Life and the evolution of Earth’s atmosphere. Science.

[13] Kaushik N., Mitra S., Baek E.J., Nguyen L.N., Bhartiya P., Kim J.H., Choi E.H., Kaushik N.K. (2023). The inactivation and destruction of viruses by reactive oxygen species generated through physical and cold atmospheric plasma techniques: Current status and perspectives. J. Adv. Res..

[14] Suhail S., Zajac J., Fossum C., Lowater H., McCracken C., Severson N., Laatsch B., Narkiewicz-Jodko A., Johnson B., Liebau J. (2020). Role of Oxidative Stress on SARS-CoV (SARS) and SARS-CoV-2 (COVID-19) Infection: A Review. Protein J..

[15] Hayashi K., Asai S., Umezawa K., Kakizoe H., Miyachi H., Morita M., Akaike T., Kuno H., Komatsu S., Watanabe T. (2022). Virucidal effect of monogalactosyl diacylglyceride from a green microalga, *Coccomyxa* sp. KJ, against clinical isolates of SARS-CoV-2 as assessed by a plaque assay. J. Clin. Lab. Anal..

[16] Hayashi K., Lee J.B., Atsumi K., Kanazashi M., Shibayama T., Okamoto K., Kawahara T., Hayashi T. (2019). In vitro and in vivo anti-herpes simplex virus activity of monogalactosyl diacylglyceride from Coccomyxa sp. KJ (IPOD FERM BP-22254), a green microalga. PLoS ONE.

[17] Tang L., Qiu L., Liu C., Du G., Mo Z., Tang X., Mao Y. (2019). Transcriptomic Insights into Innate Immunity Responding to Red Rot Disease in Red Alga Pyropia yezoensis. Int. J. Mol. Sci..

[18] Botos I., Wlodawer A. (2003). Cyanovirin-N: A sugar-binding antiviral protein with a new twist. Cell. Mol. Life Sci..

[19] Gustafson K.R., Sowder R.C., Henderson L.E., Cardellina J.H., McMahon J.B., Rajamani U., Pannell L.K., Boyd M.R. (1997). Isolation, primary sequence determination, and disulfide bond structure of cyanovirin-N, an anti-HIV (human immunodeficiency virus) protein from the cyanobacterium Nostoc ellipsosporum. Biochem. Biophys. Res. Commun..

[20] Bouslama L., Hayashi K., Lee J.B., Ghorbel A., Hayashi T. (2011). Potent virucidal effect of pheophorbide a and pyropheophorbide a on enveloped viruses. J. Nat. Med..

[21] Shih S.R., Tsai K.N., Li Y.S., Chueh C.C., Chan E.C. (2003). Inhibition of enterovirus 71-induced apoptosis by allophycocyanin isolated from a blue-green alga Spirulina platensis. J. Med. Virol..

[22] Zainuddin E., Mundt S., Wegner U., Mentel R. (2002). Cyanobacteria a potential source of antiviral substances against influenza virus. Med. Microbiol. Immunol..

[23] Li H.C., Yang C.H., Lo S.Y. (2021). Hepatitis C Viral Replication Complex. Viruses.

[24] Stadnichuk I.N., Rakhimberdieva M.G., Bolychevtseva Y.V., Yurina N.P., Karapetyan N.V., Selyakh I.O. (1998). Inhibition by glucose of chlorophyll and phycocyanobilin biosynthesis in the unicellular red alga Galdieria partita at the stage of coproporphyrinogen III formation. Plant Sci..

[25] Xia M., Fu D., Chakraborty R., Singh R.P., Terry N. (2019). Enhanced crude oil depletion by constructed bacterial consortium comprising bioemulsifier producer and petroleum hydrocarbon degraders. Bioresour. Technol..

[26] Montes D’Oca M.G., Viêgas C.V., Lemões J.S., Miyasaki E.K., Morón-Villarreyes J.A., Primel E.G., Abreu P.C. (2011). Production of FAMEs from several microalgal lipidic extracts and direct transesterification of the Chlorella pyrenoidosa. Biomass Bioenergy.

[27] Lopez Y., Soto S.M. (2019). The Usefulness of Microalgae Compounds for Preventing Biofilm Infections. Antibiotics.

[28] Fernandes A.S., Petry F.C., Mercadante A.Z., Jacob-Lopes E., Zepka L.Q. (2020). HPLC-PDA-MS/MS as a strategy to characterize and quantify natural pigments from microalgae. Curr. Res. Food Sci..

[29] Ahmad I., Wilson D.W. (2020). HSV-1 Cytoplasmic Envelopment and Egress. Int. J. Mol. Sci..

[30] Payne S., Payne S. (2017). Chapter 34—Family Herpesviridae. Viruses.

[31] Giugliano R., Buonocore C., Zannella C., Chianese A., Palma Esposito F., Tedesco P., De Filippis A., Galdiero M., Franci G., de Pascale D. (2021). Antiviral Activity of the Rhamnolipids Mixture from the Antarctic Bacterium Pseudomonas gessardii M15 against Herpes Simplex Viruses and Coronaviruses. Pharmaceutics.

[32] Taylor E.W. (2020). RNA Viruses vs. DNA Synthesis: A General Viral Strategy That May Contribute to the Protective Antiviral Effects of Selenium. Preprints.

[33] Fontaine-Rodriguez E.C., Knipe D.M. (2008). Herpes simplex virus ICP27 increases translation of a subset of viral late mRNAs. J. Virol..

[34] Weller S.K., Coen D.M. (2012). Herpes simplex viruses: Mechanisms of DNA replication. Cold Spring Harb. Perspect. Biol..

[35] Baquero E., Albertini A.A.V., Gaudin Y. (2015). Recent mechanistic and structural insights on class III viral fusion glycoproteins. Curr. Opin. Struct. Biol..

[36] Wang L., Zhao J., Nguyen L.N.T., Adkins J.L., Schank M., Khanal S., Nguyen L.N., Dang X., Cao D., Thakuri B.K.C. (2021). Blockade of SARS-CoV-2 spike protein-mediated cell–cell fusion using COVID-19 convalescent plasma. Sci. Rep..

[37] Bai Z., Cao Y., Liu W., Li J. (2021). The SARS-CoV-2 Nucleocapsid Protein and Its Role in Viral Structure, Biological Functions, and a Potential Target for Drug or Vaccine Mitigation. Viruses.

[38] Monda V., Valenzano A., Moscatelli F., Messina A., Piombino L., Zannella C., Viggiano E., Monda G., De Luca V., Chieffi S. (2016). Modifications of Activity of Autonomic Nervous System, and Resting Energy Expenditure in Women Using Hormone-Replacement Therapy. Biol. Med. (Aligarth).

[39] Stelitano D., Franci G., Chianese A., Galdiero S., Morelli G., Galdiero M. (2019). HSV Membrane Glycoproteins, Their Function in Viral Entry and Their Use in Vaccine Studies, Amino Acids, Peptides and Proteins.

[40] Selvaratnam T., Pegallapati A.K., Montelya F., Rodriguez G., Nirmalakhandan N., Van Voorhies W., Lammers P.J. (2014). Evaluation of a thermo-tolerant acidophilic alga, Galdieria sulphuraria, for nutrient removal from urban wastewaters. Bioresour. Technol..

[41] Sun Y., Shi M., Lu T., Ding D., Sun Y., Yuan Y. (2021). Bio-removal of PtCl(6)(2-) complex by Galdieria sulphuraria. Sci. Total Environ..

[42] Scherhag P., Ackermann J.U. (2021). Removal of sugars in wastewater from food production through heterotrophic growth of Galdieria sulphuraria. Eng. Life Sci..

[43] Ju X., Igarashi K., Miyashita S., Mitsuhashi H., Inagaki K., Fujii S., Sawada H., Kuwabara T., Minoda A. (2016). Effective and selective recovery of gold and palladium ions from metal wastewater using a sulfothermophilic red alga, Galdieria sulphuraria. Bioresour. Technol..

[44] Cheng F., Mallick K., Henkanatte Gedara S.M., Jarvis J.M., Schaub T., Jena U., Nirmalakhandan N., Brewer C.E. (2019). Hydrothermal liquefaction of Galdieria sulphuraria grown on municipal wastewater. Bioresour. Technol..

[45] Cheng F., Cui Z., Mallick K., Nirmalakhandan N., Brewer C.E. (2018). Hydrothermal liquefaction of high- and low-lipid algae: Mass and energy balances. Bioresour. Technol..

[46] Liu L., Sanchez-Arcos C., Pohnert G., Wei D. (2021). Untargeted Metabolomics Unveil Changes in Autotrophic and Mixotrophic Galdieria sulphuraria Exposed to High-Light Intensity. Int. J. Mol. Sci..

[47] Serive B., Nicolau E., Berard J.B., Kaas R., Pasquet V., Picot L., Cadoret J.P. (2017). Community analysis of pigment patterns from 37 microalgae strains reveals new carotenoids and porphyrins characteristic of distinct strains and taxonomic groups. PLoS ONE.

[48] Getachew P., Getachew M., Joo J., Choi Y.S., Hwang D.S., Hong Y.K. (2016). The slip agents oleamide and erucamide reduce biofouling by marine benthic organisms (diatoms, biofilms and abalones). Toxicol. Environ. Health Sci..

[49] Çelik P.A., Manga E.B., Çabuk A., Banat I.M. (2021). Biosurfactants’ Potential Role in Combating COVID-19 and Similar Future Microbial Threats. Appl. Sci..

[50] Saide A., Lauritano C., Ianora A. (2020). Pheophorbide a: State of the Art. Mar. Drugs.

[51] Meunier T., Desmarets L., Bordage S., Bamba M., Hervouet K., Rouille Y., Francois N., Decossas M., Sencio V., Trottein F. (2022). A Photoactivable Natural Product with Broad Antiviral Activity against Enveloped Viruses, Including Highly Pathogenic Coronaviruses. Antimicrob. Agents Chemother..

[52] Jimenez-Aleman G.H., Castro V., Londaitsbehere A., Gutierrez-Rodriguez M., Garaigorta U., Solano R., Gastaminza P. (2021). SARS-CoV-2 Fears Green: The Chlorophyll Catabolite Pheophorbide A Is a Potent Antiviral. Pharmaceuticals.

[53] Haarr L., Skulstad S. (1994). The herpes simplex virus type 1 particle: Structure and molecular functions. Review article. APMIS.

[54] Johnston C., Gottlieb S.L., Wald A. (2016). Status of vaccine research and development of vaccines for herpes simplex virus. Vaccine.

[55] Sanchez-Leon E., Bello-Morales R., Lopez-Guerrero J.A., Poveda A., Jimenez-Barbero J., Girones N., Abrusci C. (2020). Isolation and characterization of an exopolymer produced by Bacillus licheniformis: In vitro antiviral activity against enveloped viruses. Carbohydr. Polym..

[56] Lamers S.L., Newman R.M., Laeyendecker O., Tobian A.A.R., Colgrove R.C., Ray S.C., Koelle D.M., Cohen J., Knipe D.M., Quinn T.C. (2015). Global Diversity within and between Human Herpesvirus 1 and 2 Glycoproteins. J. Virol..

[57] Wang B., Liu Q., Huang Y., Yuan Y., Ma Q., Du M., Cai T., Cai Y. (2018). Extraction of Polysaccharide from Spirulina and Evaluation of Its Activities. Evid. Based Complement. Altern. Med..

[58] Brignati M.J., Loomis J.S., Wills J.W., Courtney R.J. (2003). Membrane association of VP22, a herpes simplex virus type 1 tegument protein. J. Virol..

[59] Chianese A., Zannella C., Monti A., De Filippis A., Doti N., Franci G., Galdiero M. (2022). The Broad-Spectrum Antiviral Potential of the Amphibian Peptide AR-23. Int. J. Mol. Sci..

