# Supplementary material for: Galdieria sulphuraria: An Extremophilic Alga as a Source of Antiviral Bioactive Compounds
Source: Mar Drugs. 2023 Jun 28;21(7):383. doi: 10.3390/md21070383 (PMC10381441; doi:10.3390/md21070383)
Supplement: Supplementary file 1 [file marinedrugs-21-00383-s001.zip › marinedrugs-2470597-supplementary.pdf]

marine drugs

MDPI

Article

Galdieria sulphuraria: an extremophilic algae as source of antiviral bioactive compounds

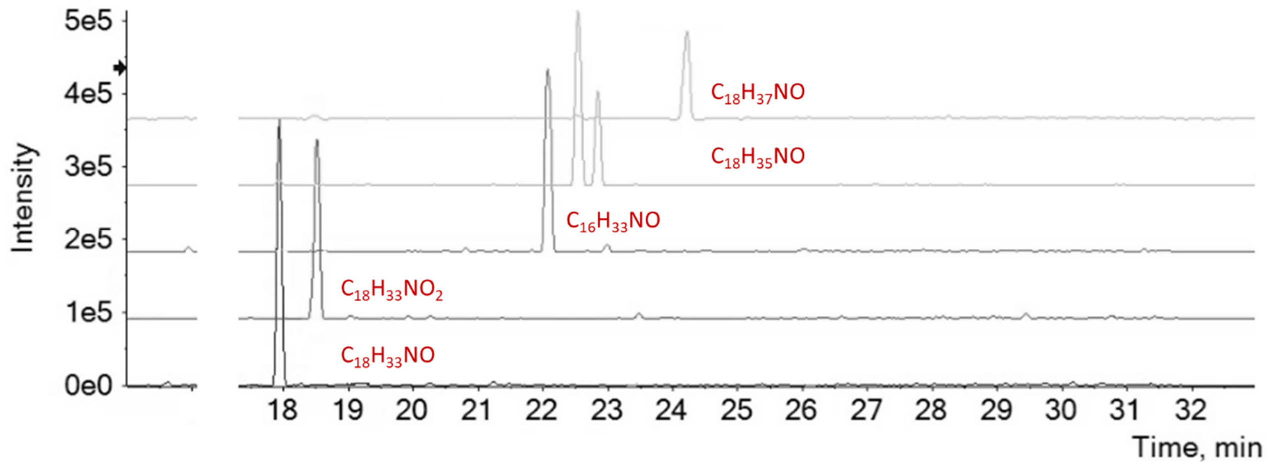

Figure S1. Extracted ion chromatograms (XICs) of detected and tentatively identified fatty acid amides.

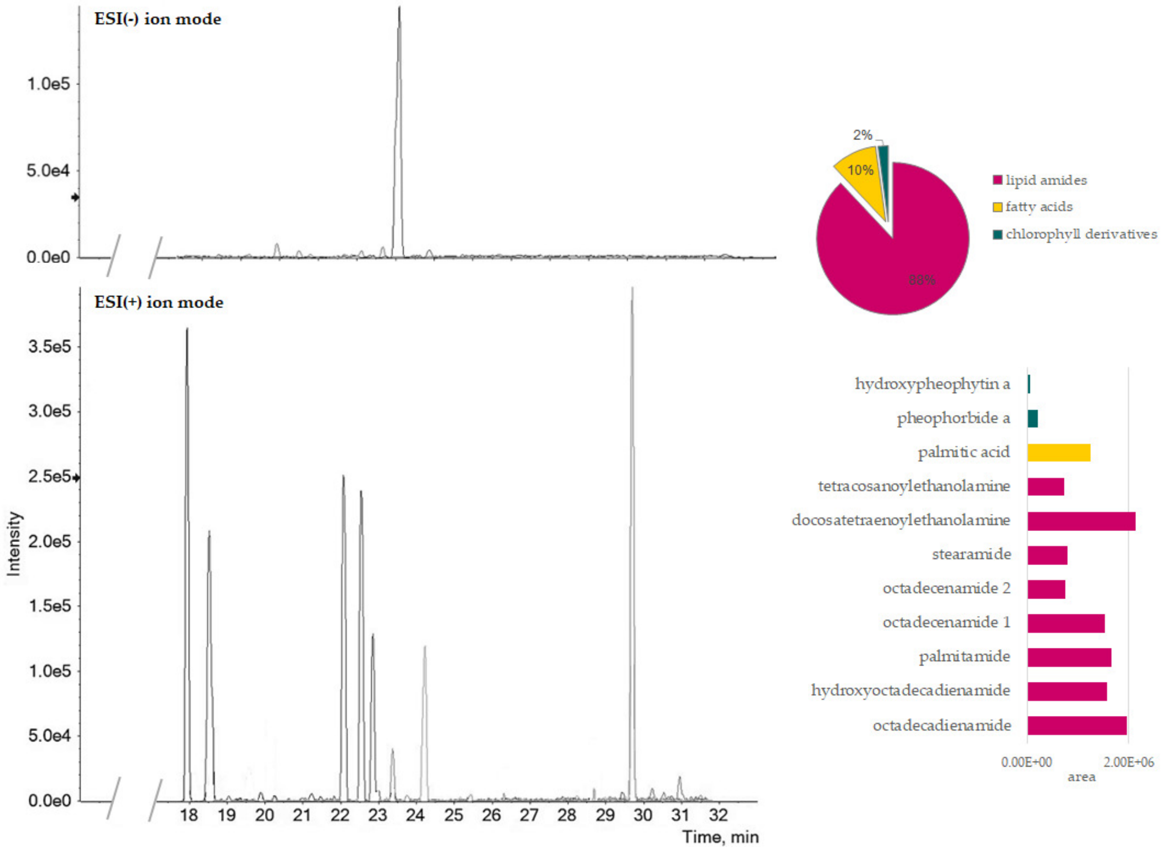

Figure S2. TICs (Total Ion Chromatograms) acquired in ESI(-) ion mode (upper panel) and ESI(+) ion mode (lower panel). Relative quantitation data are also reported.

marine drugs

MDPI

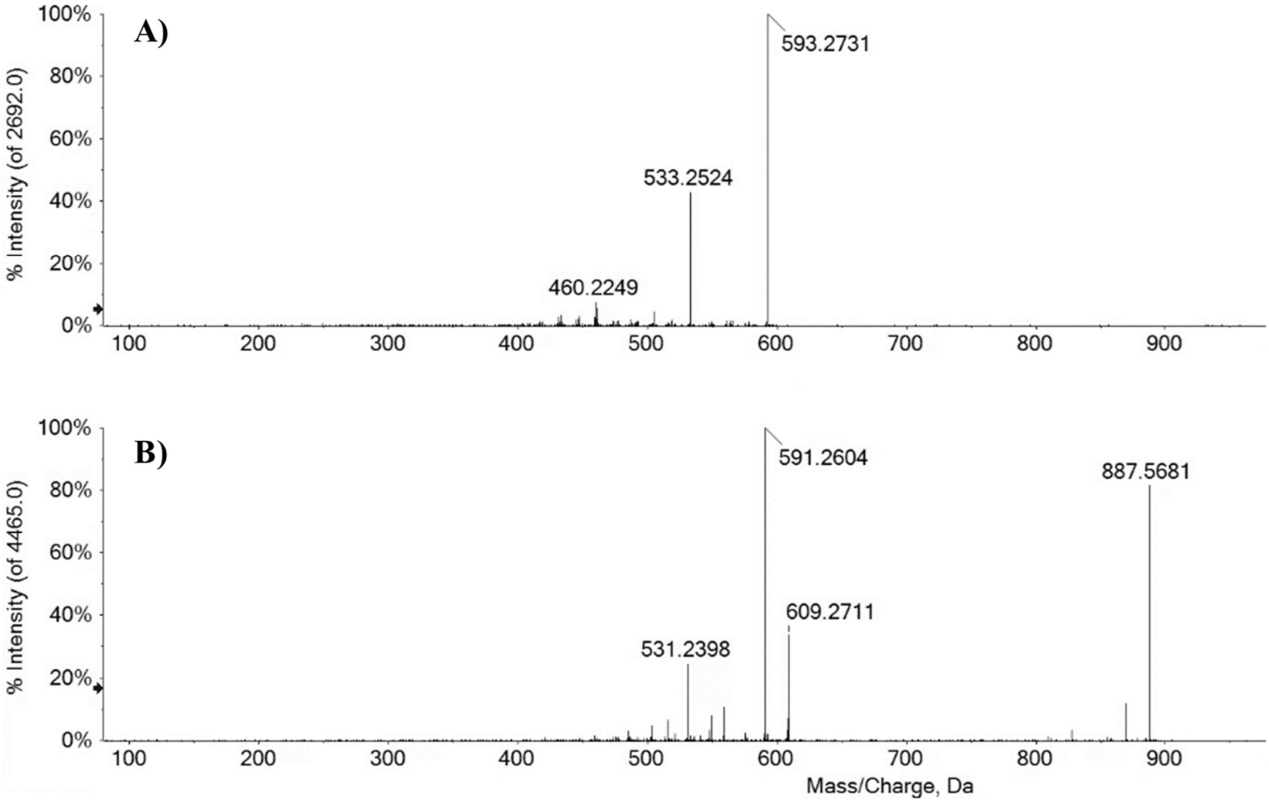

Figure S3. TOF-MS/MS spectrum of (A) pheophorbide a and (B) hydroxypheophytin a.
